# 

**Published:** 1909-06

**Authors:** 


### The Indomitable Spirit.

A CREED FOR THE DISCOURAGED.

*I believe that God created me to be happy, to enjoy the blessings of life, to be useful to my fellow beings, and an honor to my country.*

*I believe that the trials which beset me today are but the fiery tests by which my character is strengthened, ennobled and made worthy to enjoy the higher things of life which I believe are in store for me.*

*I believe that my soul is too grand to be crushed by defeat; I will rise above it.*

*I believe that I am the architect of my own fate; therefore,*

*I will be master of circumstances and surroundings, not their slave.*

*I will not yield to discouragements; I will trample them under foot and make them serve as stepping-stones to success. I will conquer my obstacles and turn them into opportunities.*

*My failures of today will help to guide me on to victory on the morrow.*

*The morrow will bring new strength, new hopes, new opportunities and new beginnings. I will be ready to meet it with a brave heart, a calm mind and an undaunted spirit. In all things I will do my best, and leave the rest to the Infinite.*

*I will not waste my mental energies by useless worry. I will learn to dominate my restless thoughts and look on the bright side of things.*

*I will face the world bravely; I will not be a coward. I will assert my God-given birthright and be a man.*

*For I am immortal and nothing can overcome me.—Virginia Opal Myers, in New Idea Woman's Magazine.*

### INDEX.

Abstracts and Selections..29, 83, 121, 176, 207, 258, 394, 435, 479, 552.
Acute Mastoiditis—Early Operation a Necessity for the Patient's Safety. By Robert E. Moss, M. D., San Antonio, Texas .......................................... 333
Acute Traumatic Tetanus Treated by Magnesium Sulphate, With Report of a Case in the Treatment of Which Injections of an Aqueous Twenty-five Per Cent Solution of Magnesium Sulphate Were Made in the Spinal Subarachnoid Space; With Recovery. By Aime Paul Heineck, Chicago, Ill..... 499
Additional Provision for the Insane. By F. S. White, Terrell, Texas.......................................... 290
A Mediocrity Up-to-Date. By Henry K. Leake, A. M., M. D., Dallas, Texas.................................. 371
Anatomic Operation for Radical Cure of Inguinal Hernia Under Local Anesthesia. By O. L. Norsworthy, Houston, Texas ............................................ 100
Annual Meeting of the Texas State Medical Association.... 463
A Tribute to the Medical Profession...................... 21
Auto-Intoxication. By R. B. Sellers, M. D., Comanche, Texas 15

Belling the Cat.......................................... 196

Books and Magazines.......................130, 216, 358, 402

Chronicles of the Octopus............................296, 338
Communications.......................................... 204
Corpus Christi Meeting of Association of Texas Health Officers 93
Correspondence................27, 78, 304, 348, 391, 430, 474

Diabetes Mellitus. By J. C. Densten, M. D., Scranton, Pa.. 327
Dr. Fly's Letter, and What Has Come of It,—So Far....... 469
Dr. M. A. Taylor, Austin, Texas......................... 254
Dr. Wainwright's Book.................................... 253

Editorialets.........24, 77, 113, 171, 203, 255, 302, 388, 429
Ethical Past of the Man at the Helm, The................ 319

Georgius Hierarchus Simmonsius—The Man in the Glass House. By G. Frank Lydston, M. D., Chicago........... 365

Important and Notable Gathering......................... 383
Indomitable Spirit, The.................................. 514
International Congress on Tuberculosis................... 111

Letters to the Editor.................................. 526
Lymphatic Cyst of the Neck—Report of a Case. By Dr. C. S. Venable, San Antonio.......................... 245

Medical Politics in Epigram and Otherwise. By G. Frank Lydston, M. D., Chicago.......................... 275
Motherhood............................................ 521

Our State Organization................................ 424
Our Unfortunate Insane. By Dr. G. I. King, Elgin, Texas.. 409

Pathology of Fear. By W. T. Marrs, M. D., Peoria, Ill...... 461
Personal Liberty. By G. B. Dorrell, M. D................ 416
Prosperine Again...................................... 247
Publisher's Notes............................84, 132, 531
Pyorrhea Alveolaris. By Julian Smith, D. D. S., Austin, Texas 166

Regulating Professional Conduct by Law................. 191
Regulation of the Social Evil in our Large American Cities. By G. Frank Lydston, M. D., Chicago................. 183
Report of the Corpus Christi Meeting of Association of Texas Health Officers.................................. 47
Retrodisplacement of the Uterus. By Harry K. Leake, M. D., Dallas, Texas.................................. 57

Sanitation of Meat Markets. By Dr. B. T. Young, Assistant State Health Officer, Austin........................ 12
Selected..................................354, 392, 433
Some Practical Remarks Anent the Proposed Texas State Tuberculous Sanitarium. By C. H. Wilkinson, M. D., Galveston, Texas...................................... 240
Some Reflections on Psychotherapy. By Dr. Julius Grinker, Chicago ............................................ 159
Some Remarks on Lacerations of the Perineum; A Plea for More Thoroughness in the Prevention, Also for the Immediate Repair; the Author's Technique. By Addison L. Lincecum, M. D., Louise, Texas....................... 413
Song of the First Lord................................. 337
Sympathy.............................................. 521

The Anti-Tuberculosis Crusade and Phthisiophobia. By Theo. Y. Hull, M. D., San Antonio, Texas.................. 453
The Auto-Protective Resources of the Body—A New Foundation for Scientific Therapeutics. By Charles E. De M. Sajous, M. D., Philadelphia........................ 137

The Fly in the Ointment.............................. 301
The Lifting of the Veil.............................. 463
The Passing of Aristocracy in Medicine............... 515
Therapeutic Nihilism: Is It Ignorance or Egotism? By H. L. Henderson, M. D., Astoria, Oregon.................. 229
The Texas Health Officers' Association.................. 1
The Tidal Wave of Sanitation......................... 23
The Trustees Can't See It............................ 202
The White Feather in Evidence........................ 419

Vital Statistics. By Dr. S. Burg, City Health Officer of San Antonio, Texas.................................... 10

Why I Write for Independent Journals. By G. Frank Lydston, Chicago, Ill.................................. 66
Women and Sanitation. By Mrs. J. M. Andrews......... 378

